# Physical Activity and Alzheimer’s Disease: A Narrative Review

**DOI:** 10.14336/AD.2019.0226

**Published:** 2019-12-01

**Authors:** Piotr Gronek, Stefan Balko, Joanna Gronek, Adam Zajac, Adam Maszczyk, Roman Celka, Agnieszka Doberska, Wojciech Czarny, Robert Podstawski, Cain C. T Clark, Fang Yu

**Affiliations:** ^1^Faculty of Physical Education, Sport and Rehabilitation, Poznan University of Physical Education, Poland.; ^2^Department of Physical Education and Sport, Faculty of Education, Jan Evangelista Purkyne University in Usti nad Labem, Czech Republic.; ^3^Department of Physical Education, University of Physical Education and Sport, Gdansk, Poland.; ^4^Department of Methodology and Statistics, The Jerzy Kukuczka Academy of Physical Education in Katowice, Katowice, Poland.; ^5^Faculty of Physical Education, Department of Human Sciences, University of Rzeszow, ul. Towarnickiego 3, 35-959 Rzeszów, Poland.; ^6^Faculty of Environmental Sciences, University of Warmia and Mazury in Olsztyn, Olsztyn, Poland.; ^7^Faculty of Health and Life Sciences, Coventry University, Coventry, CV1 5FB, United Kingdom.; ^8^School of Nursing, University of Minnesota, Minneapolis, MN 55455, USA

**Keywords:** Alzheimer’s disease, prevention, physical activity, exercise, aging

## Abstract

Although age is a dominant risk factor for Alzheimer’s disease (AD), epidemiological studies have shown that physical activity may significantly decrease age-related risks for AD, and indeed mitigate the impact in existing diagnosis. The aim of this study was to perform a narrative review on the preventative, and mitigating, effects of physical activity on AD onset, including genetic factors, mechanism of action and physical activity typology. In this article, we conducted a narrative review of the influence physical activity and exercise have on AD, utilising key terms related to AD, physical activity, mechanism and prevention, searching the online databases; Web of Science, PubMed and Google Scholar, and, subsequently, discuss possible mechanisms of this action. On the basis of this review, it is evident that physical activity and exercise may be incorporated in AD, notwithstanding, a greater number of high-quality randomised controlled trials are needed, moreover, physical activity typology must be acutely considered, primarily due to a dearth of research on the efficacy of physical activity types other than aerobic.

Through the course of aging, several processes of degradation occur in brain structure and function; commonly including; reduced blood perfusion [1], neurotransmission dysfunction [2], cortical atrophy [3], and cognitive decline [4]. Aging axiomatically predisposes older adults to Alzheimer's Disease (AD) which is one of the most prevalent neurodegenerative disorders globally [5], for which there is, currently, no cure. AD is characterized by the loss of neurons, mainly in the hippocampus and cerebral cortex, with global occurrence estimated to reach 65.7 million by 2030 [6]. According to World Health Organization (WHO) data, AD is among the top 10 causes of death in developed countries, appearing at 7th place for the first time in the top 10 causes of mortality in upper-middle-economies, and moves up to the 3rd place in high-income economies; however, in low-income and lower-middle-income countries, AD does not feature among the top 10 [7].

The prevalence and impact of AD may be partially attributable to low levels of physical activity (PA) and proclivity towards sedentary lifestyles, particularly in high-income populations. Although the literature is replete with studies presenting that systematic exercise or at least regular PA confers a favourable protective effect against some chronic disorders and the development or severity [11,12], current levels of physical inactivity are concerning. There is innumerate empirical evidence regarding the relationship between the level of physical fitness and risk of all-cause and cause-specific mortality [11], moreover, an asymptote exists between metabolic equivalents (METs) and age-adjusted mortality rates. It has been shown that mortality is independent at MET values > ~9 in women and >~10 in men but increases when below these values [11]. Therefore, low physical fitness is an important risk factor of mortality in both men and women [13]. Identical trends have been found in a group (*N*=15.000) of older veterans [14]. Thus, it is asserted that physical inactivity plays a pivotal role in the development of neurodegenerative disorders. Although age is a dominant risk factor for AD, epidemiological studies have shown that exercise may significantly decrease age-related risks for AD [15, 16, 17]. Thus, the aim of this study was to perform a narrative review on the genetic risks for AD, mechanisms of PA and the effect of exercise on cognition, and current evidence of different types of PA on AD onset or progression.

## Methods

The content of this review article is based on a narrative literature review conducted using online databases; Web of Science, PubMed and Google Scholar in the period from database inception to September 2018. In our literature review, we focused especially on the preventative effects of PA on AD onset, including genetic factors, mechanism of action and type of PA performed. Key search terms included; Alzheimer’s Disease, Early Onset, Late Onset, Cognitive Decline, Prevent*, Genetic*, Mechanism, Physical Activity, Exercise, Intervention. All key search terms were combined, using Boolean logic, such that one term relating Alzheimer’s disease, one term related to physical activity/exercise and one term related to mechanism, genetic or prevention, was searched, and subsequently, narratively reviewed.

A narrative review summarizes different primary studies from which conclusions may be drawn into an integrated interpretation [18, 19]. Results are of a qualitative rather than a quantitative nature and asserted to facilitate extended understanding within a field [20]. We adopted Greenhalgh et al.’s [21] methodological rhetoric to narrative reviews. Greenhalgh et al [21] argue that when trying to make sense of a complex concept, it is important to review literature from multiple sources and from diverse disciplines. The standard approach to critiquing a body of knowledge is to view the work across four broad perspectives: conceptual (i.e. what counts as a legitimate problem within this field); theoretical (i.e. how the things studied relate to one another in the world); methodological (i.e. how the problem is investigated); and instrumental (i.e. the tools and techniques used to understand the concept(s) more clearly in the real world). Their argument around narrative review is that, when confronted with multiple perspectives, the narrative is created through the process of having to traverse conceptual, theoretical, methodological, and instrumental boundaries to create coherence and a more integrated understanding of the topic under scrutiny. The theoretical question facing our team was whether physical activity may be beneficial in Alzheimer’s disease, and thus, the narrative review was performed accordingly, and segmented into the following section; Genetics for AD, EOAD and LOAD, mechanistic action of PA on AD, epidemiology and PA typology.

## Results and Discussion

### Genetics of AD

Preliminary evidence of the genetic aetiology of AD was proposed based on the observation that individuals affected with AD can be demarcated into one of two types: early-onset AD (EOAD), if diagnosed before the age of 65, and, late-onset AD (LOAD), if AD appears above 65 years old. Early-onset AD (EOAD) is caused by mutations in amyloid-β precursor protein (APP) located on chromosome 21, presenilin 1 (PSEN1) on chromosome 14 and closely related presenilin 2 (PSEN2) on chromosome 1. In late-onset AD (LOAD), a strong genetic component, namely, apolipoprotein E (APOE) is the most widely studied genetic risk factor and seems to be the only unequivocally accepted genetic risk factor for LOAD and recently brain-derived neurotrophic factor (BDNF) connected with AD [22, 23].

EOAD is associated with irreversible and progressive loss of memory and cognitive functions and characterized by intracerebral deposits of amyloid beta (Aß42) [24]. Aß denotes peptides of 36-43 amino acids (generated by processing of amyloid precursor protein (APP) by two proteases, ß- and γ-secretases). It is the main component of amyloid plaques in the brains of patients with AD that accumulates systematically over a long period of time and prior to symptomatic onset, and elicits undesirable effects, such as neurofibrillary tangles (NFTs) and neuronal degeneration and consequently loss of neurons [25]. The *PSEN-1* gene encodes a protein component of the gamma-secretase complex involved in the processing of the APP thus PSEN plays a very direct role in the proteolytic processing of APP [26]. Functionally, PSN-1 is involved in a numerous fundamental mechanisms of molecular pathways [27-29], regulation of β-amyloid precursor protein processing [30], regulation of transport [31], regulation of intracellular calcium homeostasis [32], stabilization of the cytoskeleton [33], which when disrupted lead to the AD. Mutations of the *PSN* lead to chromosomal instability and trisomy 21 mosaicism in AD patients [34] and consequently account for up to 50% of all familial AD cases [35]. Additionally, other candidate genes are considered as associated with EOAD as pleckstrin (*PSD2*), RUN and FYVE domain-containing protein (*RUFY1*), *TCIRG1*, and *RIN3* [36].

APOE plays a key role in the lipid homeostasis of hepatic and non-hepatic tissues since it is essential in circulation to transport of dietary lipids to the liver and adipose tissues. APOE is produced by the liver, macrophages, and in the central nervous system (CNS) [37] by astrocytes and microglia [38]. Additional functions of APOE are involved in the aging process and was asserted on the basis of experiments on animal models with Apoe knockout (KO) mice and in vitro studies [39, 40]. The APOE gene is composed of four exons, separated by three introns [35] and is located on the long arm of chromosome 19, in close proximity to the genes of apolipoprotein C-I and C-II and the low-density lipoprotein receptor (LDLR) which is in turn is located on the short arm of chromosome [24]. It is suggested that chromosome 19 play a special role in lipoprotein metabolism [41, 42].

In exon 4 of the APOE gene is observed the two single nucleotide polymorphisms (SNIPs), which give rise to the three major APOE alleles ε2, ε3, and ε4 (rs429358C>T: distinguishes ε3 and ε4, rs7412C>T: distinguishes ε3 and ε2) that encode six APOE genotypes. The ε4 allele is associated with increased risk and early age of onset of LOAD [43] but surprisingly, the frequency of allele ε4 carriers decreases with age [44].

Interestingly, carriers with one ε4 allele have 3-4 times [45] and those with two ε4 allele have 5-18 times greater risk of AD [46]. Additionally, in the context of identifying early biomarkers of AD risk, apolipoprotein J (clusterin) could be informative as part of a multi-component preclinical marker [47]. It should be emphasised that gene expression might be influenced by numerous environmental factors among them physical activity is the essential (major factor). A higher expression of certain genes (*PPARG*, *NR1H3*, *ABCA1*, *ABCG1*, CETP) has been observed even after of low-intensity exercise [47-50]. However, as to whether analogue process concerns genes involved in AD remains equivocal.

Historically, our hunter-gatherer ancestor’s daily energy expenditure (EE) related to food/water seeking activity was estimated at ~1,000-1,500 kcal/day [8]. This level of PA may have been necessary to optimize gene expression in a harsh environment [9,10]. Hence, PA seems to be a necessity for modern humans to lessen the impact of AD at a level reflective of our hunter-gatherer ancestors. Interestingly, APOE seems to moderate the relationship between aerobic exercise and cognition, with larger benefits of physical activity typically reported for APOE ε4 carriers [51-55]. Thus, physical activity may be involved in the modulation of pathogenic changes associated with AD, since APO ε4 carrying older adults in the highest tertile of physical activity had lower brain amyloid burden than those in the lowest exercise tertile, while in non-carriers there was no difference [56].

## Mechanistic Action of PA on AD

### PA potentially modifies the pathogenesis of AD

While there is no consensus on the pathogenesis of AD, extracellular β-amyloid (Aβ) disposition in the form of amyloid plaques and intracellular accumulation of neurofibrillary tangles are the two prevailing and most accepted hallmark pathology for AD. As a result, Aβ, tau, and neurodegeneration (synaptic loss and neuron death) have recently been listed as AD biomarkers to play an essential role in determining whether symptoms of dementia are truly due to AD [57]. Meanwhile, alternative theories about AD pathogenesis have been extensively investigated, including but not limited to insulin resistance and glucose hypometabolism [94], neuroinflammation [58], and oxidative stress [59]. Currently, it remains unclear whether these alternative processes are the root causes or the results of Aβ and hyperphosphorylated tau. However, it is highly plausible that AD-hallmark AD Aβ/hyperphosphorylated tau and alternative processes form a positive feedback loop. For example, the accumulation of Aβ produces more oxidative stress and more oxidative stress contributes to more amyloid accumulation.

Aβ is an endogenous protein that is produced from a transmembrane protein, amyloid precursor protein (APP) [60]. The physiological function of APP remains unclear, but may play an important role in brain development, synaptic plasticity, and neuroprotection [61]. APP is sequentially clipped first by enzyme β-secretase whose activity originates from an integral membrane aspartyl protease named β-site APP cleaving enzyme 1 (BACE-1). Next, γ-secretase, an enzyme complex including presinilin, cuts APP within the neuron’s intramembrane [61] The brains of patients with both familial and sporadic AD showed elevated BACE-1 activities which produce two major isoforms of pathogenic Aβ, the 42-residue Aβ42 and the 40-residue Aβ40. Aβ42 and 40 form fibrils which cluster into amyloid plaques [61]. Furthermore, amyloidosis is believed to be necessary for neurofibrillary tangles to occur [62]. In AD, a microtubule-associated protein, tau, becomes hyperphosphorylated due to increased activities of tau kinases and decreased activities of tau phosphatases (particularly protein phosphatase-2A), resulting in hyperphosphorylated tau. Hyper-phosphorylated tau is toxic by harboring normal tau and two other microtubule-associated proteins that compensate for the loss of tau (MAP1A/MAP1B and MAP2) to disrupt and inhibit the microtubules. The affected neurons compensate by synthesizing new normal tau and packaging phosphorylated tau into neurofibrillary tangles [63]. Exercise has been shown to reduce the accumulation of Aβ by inhibiting BACE-1 activity, enhancing the clearance of Aβ40 and 42, increasing the function of γ-secretase by releasing a large ectodomain of APP (sAPPα) that are neural and cognitive protective, and reducing tau hyperphosphorylation [61, 64].

Metabolically, impaired insulin tolerance and decreased glucose utilization have been observed in the brain of patients with AD [62]. While insulin is produced in the β-cells of the pancreas, it is transported to the brain by the cerebrospinal fluid to affect the brain physiologically or biochemically. The AD brain shows impaired insulin signaling which results in the excessive activation of glycogen synthase kinase-3β (GSK-3 β) and reduced expression of glucose transporter-1 (GLUT-1) and 3 (GLUT-3) proteins [62]. Both GSK-3 β excessive activation and reduced glucose metabolism result in hyperphosphorylated tau protein and/or Aβ plaques, abnormal nerve formation and function, and reduces cognition [65, 66]. Aerobic exercise has been shown to improve insulin signaling and glucose metabolism and promoting the production and function of insulin-like growth factors and insulin-like growth factor-binding protein 3 in animal models of AD [67, 68], as well as reduce insulin resistance in healthy older adults [69]. Human studies of healthy adults also found that 12-week high intensity interval training significantly improved glucose metabolism in the parietal-temporal and caudate regions that are often affected by AD [70].

Chronic, low-grade inflammation is common in aging and involves the chronic production of inflammatory factors such as pro-inflammatory cytokines (interleukin-6 [IL-6]) and Tumor Necrosis Factor-α [TNF-α]) in a low-grade state. Inflammation-induced production of cytokines upregulates BACE-1. Aβ deposition in turn causes topical inflammation and accelerates chronic inflammation to cause neural damage or death. Exercise has been shown to reduce the level of cytokines. In the brain, exercise has been shown to reduce IL-6 and TNF-α and reduce amyloid deposition and tau hyperphosphorylation in the brain as well as increase cognitive function in animal models [63]. Exercise in 16 weeks was shown to significantly reduce pro-inflammatory cytokines such as TNF-α in patients with mild cognitive impairment [71]

In response to exercise, there is also an increase in oxidative stress, i.e. the production of free radicals such as reactive oxygen species and reactive nitrogen species overwhelms the body’s ability to regulate and/or remove them. Free radicals originate either internally from normal essential metabolic processes as the byproducts of mitochondrial activity or from external sources such as exposure to ozone, air pollutants, and industrial chemicals. Increased production of reactive oxygen species and impaired antioxidant capacity have been observed very early in the AD pathological process including [64]. Similar to inflammation, oxidative stress also increases the production and function of BACE-1 and presinilin-1, tau hyperphosphorylation, and neurodegeneration. Aβ and hyperphosphorylated tau in turn exacerbate intracellular oxidation. Exercise has been shown to improve mitochondrial respiratory activity and reduces apoptotic signaling and neuronal death, which ultimately decrease oxidative stress in animal models with AD [72-74].

### PA stimulates plasticity of brain network

The human brain is organized into divisible functional networks during rest and varied states of activities as cognition. During performance of attention - demanding cognitive tasks, certain regions of the brain routinely increase activity, whereas others routinely decrease activity [75]. At least three brain networks; Default Mode Network (DMN), a fronto-executive network (FE), and a frontoparietal (FP) network), act on the basis of efficient communication between the frontal cortex and the rest of the brain and thus are negatively affected by aging which is associated with specific dysfunctions of brain networks [76]. The DMN is active during daydreaming and mind-wandering, auto-biographical memory, thinking, and planning for the future [77, 78] and includes hippocampal and parahippocampal, the posterior cingulate, ventral and superior frontal medial cortices, and bilateral lateral occipital, middle frontal, and middle temporal cortices [75, 77]. Increased activity of the DMN has been associated with better performance on a range of executive function tasks in older adults [79, 80], and better working memory [81]. Thus, the DMN represents an important network for understanding the determinants of healthy cognitive aging [76].

Using functional magnetic resonance imaging (fMRI) to examine low-frequency (0.008 <*f*< 0.08 Hz) coherence of cognitively relevant and sensory brain networks, longitudinally, in older adults, Voss *et al.* [76] for the first time demonstrated that aerobic training improves the aging brain’s resting functional efficiency in higher-level cognitive networks. Further, the authors [76] found strong evidence that physical activity (walking) increased functional connectivity within the DMN and a Frontal Executive Network, networks central to brain dysfunction in aging. Stretching (a non-aerobic training) and toning group also showed increased functional connectivity in the DMN after 6 months and in a Frontal Parietal Network after 12 months, possibly reflecting experience-dependent plasticity [76]. Voss et al. [76] was the first study that demonstrated the existence of exercise-induced functional plasticity in large-scale brain systems in the aging brain. Analogous results were found by Ozbeyi et al. [82] who compared varying exercise models (aerobic, resistance, combined: aerobic + resistance), and noted that exercise may have protective effects in the development stage of AD by improving the antioxidant system and brain plasticity.

### Exercise stimulates neural plasticity

Exercise upregulates expression of IGF-1 [83, 84], BDNF [85], moderates plasticity in the hippocampus and cortex, increases resting state perfusion in the hippocampus [86], increases dendritic complexity and the number of dendritic spines in the dentate gyrus [87], CA1 and entorhinal cortex (the main interface between the hippocampus and neocortex) [88, 89], increases synaptic plasticity, spatial memory and pattern separation in adult animals [90] and increases fine discrimination [91]. Studies on animal models show that aerobic exercise (voluntary wheel running) can reverse declining neurogenesis and memory function [92-94], improve pattern separation by preserving the hippocampal processing of memory details over time, which might decay rapidly otherwise [95].

However, much collective knowledge about the positive influence of aerobic exercise and environmental enrichment on improve memory and learning was inferred from a rodent-based model (often focusing on effects in the dentate gyrus (DG), a sub-region of the hippocampus). Whitemann [96] have taken a translational approach considering putative physical and neural correlates of exercise adaptation cross-sectionally in healthy young adults [96]. The authors [96] described positive influences of aerobic exercise and environmental enrichment for cognition, in particular for hippocampus-supported learning and memory, in 33 participants [96]. Further, gray matter volume in a region of the right EC composed of 157 voxels was positively associated with aerobic fitness.

Evidently, early intervention with voluntary exercise normalized hypothalamic inflammation and neuro-degeneration, as well as glucose metabolism in the 3xtg-AD animal model, suggests a hypothalamic-mediated mechanism where exercise prevents the progression of dementia and AD.

## Epidemiological evidence on the preventative effect of PA on AD risk

The pioneering research of Spirduso and Clifford [97] highlighted that older athletes’ performance on comparable tasks was substantially better than the older sedentary adults, and, indeed, was similar to the performance of the young sedentary adults. Jedrziewski et al. [98] found that aerobic exercise decreased the risk of cognitive impairment at 10-year follow-up [98]. It is asserted that exercise enhances hippocampal neurogenesis [99, 100] and cognitive function especially learning in aging [101].

## Current evidence on the effects of different types of exercise in AD

There is strong, empirical evidence that inactivity, obesity, and insulin resistance are significant risk factors for the development of AD [114]. Whilst there is considerable evidence that aerobic training, such as running and dancing (and probably other types of aerobic training), may lower the risk of AD, notwithstanding, there is a paucity of evidence that dynamic resistance training or static resistance training lowers risk of AD. Nevertheless, Parise et al. [115] examined 28 older men and women (68.5 ± 5.2y) who performed whole-body resistance exercise training (RET) for 14 weeks and analysed of muscle biopsies taken before and 72 h following the last exercise bout from the Vastus Lateralis. Parise et al. [115] observed that RET was associated with a decrease in 8-OHdG (Pre: 10783+/-5856, Post: 8897+/-4030 ng g(-1) creatinine; p<0.05); and complex IV activity was significantly higher after training as compared to before training (Pre: 2.2+/-0.5, Post: 2.9+/-0.9 micromol min(-1) g(-1) ww; p<0.05), as was the ratio of complex IV to complex I (Pre: 11.1+/-9.3, Post: 14.5+/-10.3; p<0.05). These results suggest that regular RET decreases oxidative stress, moreover, increases in complex IV of the electron transport chain may have an indirect antioxidant effect in older adults and may improve function in daily activities [115].

Regarding the effect of resistance training (6 months, 3 days a week, for 1 hour, 10-minute cycle ergometer warm up and stretching exercises, followed by training protocol - 6 exercises, 2 sets, 8 repetition, intensity at 50% 1RM in one experimental group and in the other at 80% 1RM, the control group practiced the same protocol, but without any load) in elderly participants (65-75y), on serum IGF-1 concentration and cognitive performance, Cassilhas et al. [116] observed increased serum IGF-1, and better cognitive performance when compared to the control group.

Resistance training for the lower limbs in non-frail and pre-frail 48 elderly women evaluated by Coelho et al. [117] showed increased serum BDNF (BDNF is associated with neuroprotection in a series of central nervous system diseases in older age) concentration after training (before 351±68 pg/ml and after 593±79 pg/ml; p<0.001). In addition, there was a significant increase in Timed Up-and-Go (TUG) test (where a subject must stand up from a seated position on a chair, walk three meters at a comfortable pace, turn around, walk back to the chair and sit down) performance. Analogous results were found in Liu-Ambrose at al. [118] and Hauer et al. [119]. Summarizing, Portugal et al. [120] analysed possible biological mechanisms related to strength training effects on the brain, and concluded that there is [currently] little evidence to indicate that strength training is a valid intervention to increase neuroprotective factor levels and thereby improve biological mechanisms associated with aging and AD. Contradictorily, Hurley et al. [121] asserted there is no evidence that resistance training can reverse any of the primary biological or behavioural outcomes of AD, however, the authors concede that there is evidence that the prevalence of this disease is inversely associated with muscle mass and strength [121].

Interestingly, dancing-based physical activity appears correlated with a decreased risk of developing dementia. Porat et al [122], whom examined 48 mild cognitive impairment (MCI) elderly participants, and identified themselves as either dancers or non-dancers, found that, despite significantly thinner cortex, dancers performed better in cognitive tasks, such as the California Verbal Learning Test-II (CVLT-II) short delay free recall (p = 0.004), the CVLT-II long delay free recall (p = 0.003), and the CVLT-II learning over trials 1-5 (p = 0.001) [122]. Musical ability is frequently positively correlated with verbal, spatial, and emotional intelligence [123, 124]; yet understanding how playing a musical instrument impacts the nervous system and how significantly this influences process on CNS older persons requires further investigation [125, 126].

### Conclusion

In conclusion, current evidence highlights that physical activity and exercise may be used in a preventative or mitigating manner for AD, and thus, should be more intensively researched using randomized controlled trials to confirm the veracity of such assertions. Notwithstanding, PA typology must also be acutely considered in order to confer any potential benefits.
